# Supplementary material for: Pyruvate Dehydrogenase Kinase 1 inhibition mediated oxidative phosphorylation enhancement in cartilage promotes osteoarthritis progression
Source: BMC Musculoskelet Disord. 2023 Jul 20;24:597. doi: 10.1186/s12891-023-06585-6 (PMC10357736; doi:10.1186/s12891-023-06585-6)
Supplement: Supplementary file 1 — Additional file 1. [file 12891_2023_6585_MOESM1_ESM.zip › KEGG.Gsea.1653622432464/KEGG_SPHINGOLIPID_METABOLISM.html]

Details for gene set KEGG\_SPHINGOLIPID\_METABOLISM[GSEA]

|  || Dataset | 55457\_collapsed\_to\_symbols.cls.cls #OA\_versus\_normal.cls.cls #OA\_versus\_normal\_repos |
| Phenotype | cls.cls#OA\_versus\_normal\_repos |
| Upregulated in class | OA |
| GeneSet | KEGG\_SPHINGOLIPID\_METABOLISM |
| Enrichment Score (ES) | 0.29911456 |
| Normalized Enrichment Score (NES) | 1.0897506 |
| Nominal p-value | 0.32779098 |
| FDR q-value | 0.48962623 |
| FWER p-Value | 1.0 |
Table: GSEA Results Summary

  

Fig 1: Enrichment plot: KEGG\_SPHINGOLIPID\_METABOLISM      
 Profile of the Running ES Score & Positions of GeneSet Members on the Rank Ordered List

  

| SYMBOL | TITLE | RANK IN GENE LIST | RANK METRIC SCORE | RUNNING ES | CORE ENRICHMENT || 1 | SGPP1 | sphingosine-1-phosphate phosphatase 1 [Source:HGNC Symbol;Acc:HGNC:17720] | 278 | 0.766 | 0.0471 | Yes |
| 2 | GLB1 | galactosidase beta 1 [Source:HGNC Symbol;Acc:HGNC:4298] | 337 | 0.731 | 0.1082 | Yes |
| 3 | GALC | galactosylceramidase [Source:HGNC Symbol;Acc:HGNC:4115] | 478 | 0.669 | 0.1573 | Yes |
| 4 | SMPD2 | sphingomyelin phosphodiesterase 2 [Source:HGNC Symbol;Acc:HGNC:11121] | 821 | 0.564 | 0.1812 | Yes |
| 5 | PLPP1 | phospholipid phosphatase 1 [Source:HGNC Symbol;Acc:HGNC:9228] | 1109 | 0.494 | 0.2032 | Yes |
| 6 | SMPD1 | sphingomyelin phosphodiesterase 1 [Source:HGNC Symbol;Acc:HGNC:11120] | 1230 | 0.471 | 0.2361 | Yes |
| 7 | B4GALT6 | "beta-1,4-galactosyltransferase 6 [Source:HGNC Symbol;Acc:HGNC:929]" | 1500 | 0.429 | 0.2537 | Yes |
| 8 | SGMS1 | sphingomyelin synthase 1 [Source:HGNC Symbol;Acc:HGNC:29799] | 1945 | 0.367 | 0.2521 | Yes |
| 9 | ASAH1 | N-acylsphingosine amidohydrolase 1 [Source:HGNC Symbol;Acc:HGNC:735] | 2201 | 0.338 | 0.2627 | Yes |
| 10 | SPHK2 | sphingosine kinase 2 [Source:HGNC Symbol;Acc:HGNC:18859] | 2478 | 0.305 | 0.2686 | Yes |
| 11 | CERK | ceramide kinase [Source:HGNC Symbol;Acc:HGNC:19256] | 2633 | 0.287 | 0.2823 | Yes |
| 12 | SPTLC1 | serine palmitoyltransferase long chain base subunit 1 [Source:HGNC Symbol;Acc:HGNC:11277] | 2913 | 0.259 | 0.2839 | Yes |
| 13 | GBA | glucosylceramidase beta [Source:HGNC Symbol;Acc:HGNC:4177] | 3327 | 0.219 | 0.2714 | Yes |
| 14 | ARSA | arylsulfatase A [Source:HGNC Symbol;Acc:HGNC:713] | 3334 | 0.218 | 0.2905 | Yes |
| 15 | SGPL1 | sphingosine-1-phosphate lyase 1 [Source:HGNC Symbol;Acc:HGNC:10817] | 3462 | 0.206 | 0.2991 | Yes |
| 16 | GLA | galactosidase alpha [Source:HGNC Symbol;Acc:HGNC:4296] | 4172 | 0.140 | 0.2566 | No |
| 17 | NEU1 | neuraminidase 1 [Source:HGNC Symbol;Acc:HGNC:7758] | 4486 | 0.114 | 0.2425 | No |
| 18 | NEU2 | neuraminidase 2 [Source:HGNC Symbol;Acc:HGNC:7759] | 5775 | 0.010 | 0.1433 | No |
| 19 | UGT8 | UDP glycosyltransferase 8 [Source:HGNC Symbol;Acc:HGNC:12555] | 5908 | 0.002 | 0.1332 | No |
| 20 | SMPD3 | sphingomyelin phosphodiesterase 3 [Source:HGNC Symbol;Acc:HGNC:14240] | 6533 | -0.045 | 0.0888 | No |
| 21 | GAL3ST1 | galactose-3-O-sulfotransferase 1 [Source:HGNC Symbol;Acc:HGNC:24240] | 8378 | -0.184 | -0.0380 | No |
| 22 | DEGS1 | "delta 4-desaturase, sphingolipid 1 [Source:HGNC Symbol;Acc:HGNC:13709]" | 8885 | -0.223 | -0.0573 | No |
| 23 | NEU3 | neuraminidase 3 [Source:HGNC Symbol;Acc:HGNC:7760] | 9348 | -0.259 | -0.0699 | No |
| 24 | SPTLC2 | serine palmitoyltransferase long chain base subunit 2 [Source:HGNC Symbol;Acc:HGNC:11278] | 9534 | -0.277 | -0.0595 | No |
| 25 | PLPP3 | phospholipid phosphatase 3 [Source:HGNC Symbol;Acc:HGNC:9229] | 9906 | -0.312 | -0.0603 | No |
| 26 | PLPP2 | phospholipid phosphatase 2 [Source:HGNC Symbol;Acc:HGNC:9230] | 11160 | -0.452 | -0.1171 | No |
| 27 | KDSR | 3-ketodihydrosphingosine reductase [Source:HGNC Symbol;Acc:HGNC:4021] | 11473 | -0.498 | -0.0967 | No |
| 28 | SPHK1 | sphingosine kinase 1 [Source:HGNC Symbol;Acc:HGNC:11240] | 12320 | -0.682 | -0.1012 | No |
| 29 | UGCG | UDP-glucose ceramide glucosyltransferase [Source:HGNC Symbol;Acc:HGNC:12524] | 12532 | -0.763 | -0.0491 | No |
| 30 | SMPD4 | sphingomyelin phosphodiesterase 4 [Source:HGNC Symbol;Acc:HGNC:32949] | 12702 | -0.866 | 0.0154 | No |
Table: GSEA details [plain text format]

  

Fig 2: KEGG\_SPHINGOLIPID\_METABOLISM      
 Blue-Pink O' Gram in the Space of the Analyzed GeneSet

  

Fig 3: KEGG\_SPHINGOLIPID\_METABOLISM: Random ES distribution      
 Gene set null distribution of ES for **KEGG\_SPHINGOLIPID\_METABOLISM**
